# Comparison of simple and complex liver intensity modulated radiotherapy

**DOI:** 10.1186/1748-717X-5-115

**Published:** 2010-11-30

**Authors:** Mark T Lee, Thomas G Purdie, Cynthia L Eccles, Michael B Sharpe, Laura A Dawson

**Affiliations:** 1 Radiation Medicine Program, Princess Margaret Hospital, University of Toronto, Toronto, Ontario, Canada; 2 Radiation Oncology Department, Peter MacCallum Cancer Centre, East Melbourne, Victoria, Australia; 3 CRUK/MRC Gray Institute for Radiation Oncology and Biology, University of Oxford, Oxford Cancer Centre, Churchill Hospital, Oxford, UK

## Abstract

**Background:**

Intensity-modulated radiotherapy (IMRT) may allow improvement in plan quality for treatment of liver cancer, however increasing radiation modulation complexity can lead to increased uncertainties and requirements for quality assurance. This study assesses whether target coverage and normal tissue avoidance can be maintained in liver cancer intensity-modulated radiotherapy (IMRT) plans by systematically reducing the complexity of the delivered fluence.

**Methods:**

An optimal baseline six fraction individualized IMRT plan for 27 patients with 45 liver cancers was developed which provided a median minimum dose to 0.5 cc of the planning target volume (PTV) of 38.3 Gy (range, 25.9-59.5 Gy), in 6 fractions, while maintaining liver toxicity risk <5% and maximum luminal gastrointestinal structure doses of 30 Gy. The number of segments was systematically reduced until normal tissue constraints were exceeded while maintaining equivalent dose coverage to 95% of PTV (PTVD95). Radiotherapy doses were compared between the plans.

**Results:**

Reduction in the number of segments was achieved for all 27 plans from a median of 48 segments (range 34-52) to 19 segments (range 6-30), without exceeding normal tissue dose objectives and maintaining equivalent PTVD95 and similar PTV Equivalent Uniform Dose (EUD(-20)) IMRT plans with fewer segments had significantly less monitor units (mean, 1892 reduced to 1695, p = 0.012), but also reduced dose conformity (mean, RTOG Conformity Index 1.42 increased to 1.53 p = 0.001).

**Conclusions:**

Tumour coverage and normal tissue objectives were maintained with simplified liver IMRT, at the expense of reduced conformity.

## Background

Conformal liver radiotherapy (CRT) has an emerging role in treating unresectable primary or metastatic cancer in the liver. Conventional and hypofractionated stereotactic body radiotherapy (SBRT) results in low reported rates of toxicity and high tumour control rates for both primary and metastatic liver cancer[1-3]. The majority of trials have treated small lesions typically <5 cm in size; however treatment of larger, multifocal tumours can be performed safely as long as doses are individualized to avoid liver and other normal tissue toxicity[1-3]. CRT planning for large multifocal tumours is challenging, and intensity modulated radiotherapy (IMRT) has the potential for improving the treatment of liver cancer, by facilitating dose escalation particularly for large tumours and/or reducing dose to normal tissues[4,5]. However the potential improvements in plan quality have to be considered against the potential drawbacks of more complex radiotherapy plans including increasing requirements for quality assurance checks and risks of errors in treatment delivery.

IMRT can improve radiation plan quality by use of mathematical and biological cost function algorithms to optimize the planned radiation dose distributions, not easily performed using non-automated forward planned segmented CRT[6]. Liver IMRT planning studies have typically used complex highly modulated plans with the number of segments used up to 100 segments per beam or 10 intensity levels[5,7,8]. However, increasing IMRT radiation fluence complexity is not beneficial for all liver cancers, and treatment of smaller lesions may benefit more from increasing plan conformity with the use of many beams and beam angles, as opposed to increasing IMRT modulation complexity[9].

The dosimetric benefit of IMRT plans with increasing number of beam segments appears to have a ceiling, with prior studies on non-liver IMRT showing reducing gains when using more than 5-9 segments per beam[10] or more than 5 intensity levels[11]. More complex highly segmented or modulated IMRT plans have the potential disadvantages of; 1) delivery of more treatment monitor units (MUs) with a resulting increase in treatment time (presuming a constant machine dose rate)[12]; 2) increased leaf leakage with potentially increased risks of second malignancy[13,14]; 3) increased sensitivity to geometric uncertainties and; 4) decreased dosimetric accuracy of IMRT delivery with potentially more time needed for accurate dosimetric quality assurance[14]. That is, more complex IMRT modulated plans may result in larger differences between the nominal and delivered doses for tumour and normal tissues. Thus, there is motivation to reduce complexity of IMRT plans if safely possible.

Image guided radiotherapy (IGRT) and motion management strategies can reduce the residual geometric uncertainties (and improve the concordance between the nominal and delivered doses). However IGRT solutions are not always available and residual geometric error will always exist, for example due to intra-fraction organ motion, deformation, change in position of organs, tolerance levels for repositioning patients, etc[15]. Dosimetric inaccuracy with radiation planning can result in up to 13% underestimation of dose delivered occurring in highly modulated regions of an IMRT field,[16] and single beam daily dose variations in the order of 15 to 35% in the presence of breathing motion, potentially resulting in systematic errors in dose calculations[17,18]. More complex IMRT plans with low weighted segments (e.g. 10-15 MUs per segment) and more segments are more susceptible to these effects,[19] with a potentially larger clinical impact for hypofractionated radiotherapy[18].

Due to the increased uncertainties that exist between the nominal and delivered doses for more complex IMRT plans than less complex plans, a strong rationale exists to investigate and use IMRT plans with simple modulation if more complex plans do not significantly improve the plan quality. Thus, a planning study was developed to simplify the radiation modulation pattern by using fewer beam segments in liver IMRT. The aim of this study was to determine the minimum number of planned segments in liver IMRT plans that could maintain adequate target coverage and normal tissue dose objectives.

## Methods

### Study Design

The primary objective was to determine if more than 80% of IMRT plans could be simplified, by using 30 or less beam segments, without a clinically significant compromise of target coverage or normal tissue sparing. A sample size of 27 cases was required to obtain an exact 95% confidence interval that more than 80% of simple IMRT plans would be acceptable (i.e. in 25 or more of 27 cases, the simplified IMRT plans would be clinically acceptable and meet tumor coverage and normal tissue sparing guidelines). This was deemed to be a clinically significant number of plans in which we would recommend treating patients with an IMRT plan using simpler beam modulation as compared to a plan with complex beam modulation. Secondary objectives were to assess the changes in number of MUs delivered, target dose conformity, and differences in doses delivered to normal structures, compared to index IMRT plans using many segments.

Plans were developed from 27 planning CT datasets from patients previously treated on a research ethics board approved phase I and II clinical trials for primary and metastatic liver cancer, of 6 fraction, individualized CRT treated with daily IGRT[20]. Unlike most SBRT experience, these studies allowed patients with large and multifocal cancers to be treated, and the prescription dose was often limited by normal tissue tolerances[2,3,20]. Initially 10 patients were selected from a patient cohort in which dosimetric benefits of IMRT compared to CRT were previously demonstrated[4]. Another 17 cases with prescription doses limited by risk of normal tissue toxicity (adjacent luminal structures or liver toxicity) were also included, as they were the types of liver cases previously found to most likely to benefit from IMRT (vs. CRT)[4].

### Index IMRT planning

An index, segmented IMRT plan was generated and evaluated for each case using Pinnacle, version 8.0, treatment planning system (ADAC, Milpitas, CA). Plan optimization was performed using direct machine parameter optimization (DMPO), a method of direct aperture optimization that allows the maximum number of IMRT segments to be specified prior to plan optimisation[21]. The index IMRT plans were optimized to have a maximum of 50 segments, although 1 plan had up to 52 segments as an optimized plan from the initial IMRT planning cohort [4]. The index IMRT plan was determined as the one that delivered the highest minimum dose to 0.5 cc of the PTV while maintaining normal tissues constraints resulting in some index plans having fewer than 50 segments.

An exhale breath-hold helical CT was used for treatment planning, and presumption of use of a breath-hold device during treatment was made for the purposes of this study, to remove the potential adverse impact of breathing motion. Gross tumour volumes (GTVs) and organs at risk (OARs) (e.g. liver, oesophagus, stomach, duodenum, bowel, heart, ribs and spinal cord) were delineated on the exhale breath-hold CT scan. A uniform 5 mm expansion around the GTVs was used to create the planning target volume (PTV)[15].

The index IMRT plans had more than 30 beam segments (maximum 52) and used 3 to 8 beams (median 5) with up to 2 non-coplanar beam angles. Individualized beam angles similar to those used in the clinical radiation plan (chosen by experienced planners) were used. These beam angles were typically chosen to spare the maximum volume of normal liver irradiated to minimise the risk of radiation induced liver disease, however other beam angles are also chosen to create steep radiation gradients near adjacent normal visceral structures. A minimum segment area of 2 cm^2 ^with a segment width of 1 cm and 10 MUs per segment were specified. Beam energies of 6 MV or 10 MV, a 2.5 mm dose grid and a convolution/superposition algorithm for dose calculation were used.

The radiation dose prescription was individualized between patients and was based on the dose covering 95% of the PTV (PTVD95). Plans were optimized to provide the highest, minimum dose covering 0.5 cc of the PTV while maintaining normal structure dose constraints (table 1). A maximum prescription dose of 60 Gy for metastases and 54 Gy for primary HCC, in 6 fractions, was specified. Hot spots were limited to 120% outside PTV and 140% inside the PTV. A maximum liver normal tissue complication probability (NTCP) of 5% was permitted, based on the Lyman-Kutcher-Burman (LKB) NTCP model[22,23], using parameters based on the data published from the University of Michigan[24], with biological corrections for dose per fraction using an α/β correction of 2.5 Gy[20].

**Table 1:** Six Fraction IMRT Planning Dose Limits

Structure	Dose Limit
Liver	5% Normal Tissue Complication Probability

Esophagus	Maximum 30 Gy to 0.5 cc

Stomach	Maximum 30 Gy to 0.5 cc

Bowel	Maximum 30 Gy to 0.5 cc

Cord	Maximum 25 Gy

Ribs	Maximum 48 Gy to 0.5 cc

Heart	Maximum 45 Gy to 0.5 cc

Kidney	Mean kidney doses <10.8 Gy

Target Volume	Maximum dose 140% of prescription

Generalized equivalent uniform dose (gEUD) was used for plan optimization to limit the dose received by the liver[25]. The gEUD also allows heterogeneous doses within a normal structure or volume to be represented as a single equivalent dose which can be weighted differently towards the maximum or minimum dose delivered to a structure. It is useful in analysing heterogeneous dose distribution over a target particularly in the setting of IMRT or conformal radiotherapy plans. To assess the effect of heterogeneous doses within the PTV, an equivalent uniform dose with an 'a' value of -20 was used (EUD(-20)), this value would reflect an aggressive tumour that would be more sensitive to low doses within the PTV[4,6].

### Reduced Segment (Simple) IMRT

**Figure 1:**
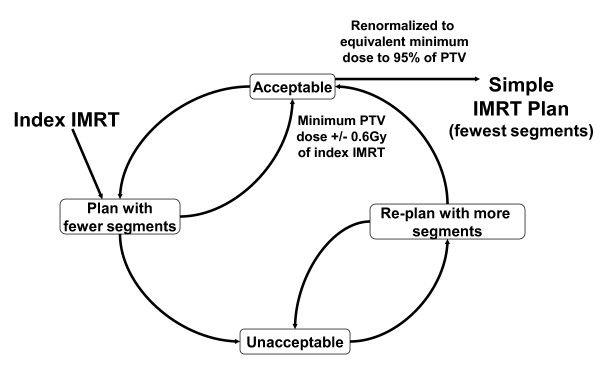
Schematic of Intensity Modulated Radiotherapy Segment Number Reduction.

The total number of segments used in the IMRT optimization was systematically reduced to the minimum number which resulted in an "acceptable" simple IMRT plan based on the criteria from table 1, while maintaining a minimum dose to 0.5 cc of PTV, within 0.6 Gy of the index IMRT plan. This acceptance criteria was chosen to be consistent with dosimetric accuracy estimated to be 2% of a 30 Gy plan (for a variation of 0.6 Gy) (Figure 1). The number of segments was altered with each re-optimization of IMRT. All plans were then renormalized to the minimum dose covering 95% of the index IMRT, and they had to meet the normal tissue constraints specified. Otherwise a plan with more segments was chosen as the simplest acceptable IMRT plan.

### Evaluation

Number of segments and treatment MUs were compared between simple and index IMRT plans. Plan conformity was assessed using the RTOG conformity index (RTOG CI), we defined the relevant doses as the quotient of the total isodose volume of the minimum dose covering 0.5 cc of the PTV on the index plan and volume of the PTV. Isodose volumes outside of the PTV were compared, as a measure of high dose (42 Gy), moderate dose (30 Gy) and low dose (1 Gy) conformity. Additionally integral dose was assessed as the joules (J) received to the treatment volume outside of the PTV based on the calculated doses from the treatment planning system. Assumptions were made that the density of tissue in the irradiated volume was 1 g/mL and is used to compare the potential impact of lower doses delivered to larger volumes by more complicated modulated radiotherapy.

Summary statistics were analyzed for minimum PTV dose to 99% (PTVD99), PTVD95, 0.5 cc of the PTV and the PTV EUD (-20). Normal liver (liver minus GTV), rib, heart, spinal cord and gastrointestinal visceral structure maximum and mean doses were assessed. The effective liver volume (Veff) irradiated was used as a measure of volume of normal liver irradiated[22].

A novel in-house complexity metric for IMRT was also used to assess plans[26]. In brief, this complexity metric is calculated based on three parameters that are independent of the segment number: the relative segment weight, the segment area and the position of the multi-leaf collimator (MLC) defining the segment shape. A simple plan would have the least amount of beam modulation (i.e. one open beam aperture) with the highest score of 1, and a complex IMRT plan would have a low score closer to 0. This metric is associated with the dosimetric accuracy between the planned and actual delivered dose of an IMRT plan, as reported by McNiven et al. [26]

### Statistics

Analyses were performed using SPSS version 16. Wilcoxon signed ranked tests were performed to test for statistical differences in doses for the primary outcomes. Exploratory analysis and secondary outcomes was performed using forward multivariate linear regression, Mann-Whitney and Kruskal-Wallis tests for non-parametric data. For statistical analysis, a two sided p-value of <0.05 was considered significant. No corrections for multiple analyses were performed for secondary analyses. P values were used to help indicate possible associations of between IMRT plans and dose relationships.

## Results

### Tumour/planning characteristics

Eleven patients with primary liver cancer and sixteen with liver metastases having a total of 45 liver tumours (tumours per patient range: 1 - 5) were included in this planning study. Dose escalation was limited by adjacent OARs and/or risk of liver toxicity in 24 of the 27 patients (table 2).

**Table 2:** Patient Characteristics

	Total	Dose Limiting Structure			
		
		None	Liver	Non-Liver OARs	Liver & other OARs
Number	27	3	5	10	9

Diagnosis					
Metastases	17	2	1	9	5
Hepatocellular	10	1	4	1	4

Tumor Number					
Mean	1.7	1	1.6	2	1.6
Range	1-5	1	1-3	1-5	1-3

Tumor Volume, cc					
Mean	211.6	11	283	150.4	306.6
Range	4.2-756	6.7-13.8	16.9-707	4.2-394.1	52.4-756

Prescription, Gy					
Mean	42.4	57	42.9	39.4	40.5
Range	28.1-60	55.3-60	32.6-55.3	28.8-54.4	28.1-55.3

In all patients, an "acceptable" simple IMRT plan with ≤30 beam segments was achieved without exceeding normal structure dose constraints or clinically compromising PTV coverage. The number of beam segments for the simplest acceptable IMRT plans (median 19; range: 6-30) was significantly less than number of beam segments in the index IMRT plan (median 48; range 34-52), p < 0.001. The 95% confidence interval (adjusted wald) of acceptable simple IMRT plans that could be obtained using 30 beam segments or less is 85.2% to 100%.

There was little correlation between the tumour number, volume, number of beams or index plan complexity and minimum number of IMRT segments associated with plan acceptability on multivariate analysis (maximum model R^2 ^= 0.127).

The total number of plan MUs was significantly lower with simple IMRT (mean 1695 MUs vs. 1892 MUs, p = 0.012), with significantly more MUs delivered per segment (mean 106 MUs/segment vs. 40 MUs/segment, p < 0.001).

### Target Coverage

**Figure 2:**
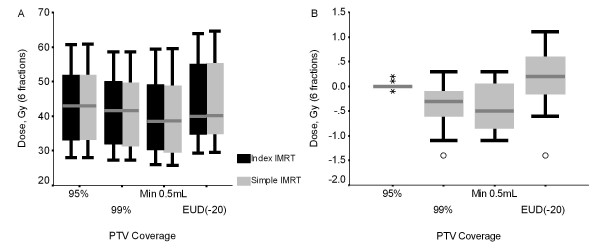
Nominal PTV dose for the index and simple IMRT plans showing the median, interquartile range (box), and range (whiskers or stars (outliers)) of doses for PTV D95, PTV D99 and PTV EUD (-20) (A) and differences of dose for index and simple IMRT.

There was no overall differences seen in the PTVD95 or PTV EUD (-20) between the simple and index IMRT plans (mean 44.6 Gy vs. 44.5 Gy, p = 0.066). As expected, the dose to 0.5 cc of the PTV was statistically less for simple IMRT compared to index IMRT (mean 39.5 Gy vs. 40.0 Gy, p0.006), as was PTVD99 (mean 40.6 Gy vs. 41 Gy, p < 0.001) (figure 2), since small differences in minimum dose to 0.5 cc of PTV were permitted in the study design and likely of little clinical significance as summarized in table 3. The maximum doses within PTV were higher for the simple IMRT compared to index IMRT (mean 123.4% vs. 121.3%, p = 0.036).

**Table 3:** Differences between Nominal Index and Simple IMRT plans

Structure	Index IMRT Mean [Range]	Simple IMRT Mean [Range]	p-value
Segment Number*	47 [34-52]	18 [6-30]	**<0.001**

Complexity Score	0.51 [0.2-0.72]	0.63 [0.35-0.85]	**<0.001**

RTOG CI	1.42 [0.99-2.02]	1.52 [1.11-2.21]	**0.001**

Monitor Units	1892 [953-3761]	1695 [934-3832]	**0.012**

PTV D95 (Gy)	42.4 [28.1-60.7]	42.4 [28.1-60.8]	0.197

PTV D99 (Gy)	41.0 [27.3-58.7]	40.6 [27.2-58.7]	**<0.001**

PTV Min. 0.5 mL (Gy)	40.0 [25.7-59.4]	39.5 [25.7-59.5]	**0.006**

PTV EUD (-20) (Gy)	44.5 [29.4-63.9]	44.6 [29.6-64.6]	0.066

Liver NTCP (%)	2.7 [0-5]	2.4 [0-5]	0.370

Effective Liver Volume	0.38 [0.07-0.7]	0.38 [0.07-0.71]	0.418

Mean Liver Dose (Gy)	15.1 [2.4-20.2]	15.1 [2.7-19.8]	0.809

Mean Kidney Dose (Gy)	3.3 [0-13.7]	3.4 [0.3-11.7]	0.509

Max. Cord Dose (Gy)	16.4 [2.6-24]	16.2 [2.3-24.8]	0.713

Max.Stomach Dose (Gy)	19.6 [1.1-30]	19.4 [0.6-30]	0.548

Max. Duodenum Dose (Gy)	10.1 [0-29.9]	9.9 [0-29.4]	0.648

Max. Bowel Dose (Gy)	9.9 [0-30]	9.7 [0-30]	0.545

Max. Esophagus Dose (Gy)	16.1 [0-30]	16.1 [0-29.6]	0.980

Max. Heart Dose (Gy)	24.5 [0-43.9]	25.3	**0.048**

Max. Ribs Dose (Gy)	37.7 [0-48]	38.2 [0-48]	**0.044**

Integral Dose (J)	68.7 [4.7-181.6]	69.1 [5.2-173.9]	**0.374**

* primary endpoint for analysis

Max. is maximum dose to 0.5 mL of relevant structure except for cord where the point maximum dose is reported.

### Normal Tissue Dose

**Figure 3:**
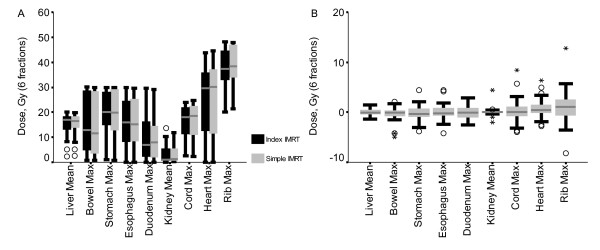
Nominal normal tissue dose for the index and simple IMRT plans showing the median, interquartile range (box), and range (whiskers or stars (outliers)) (A) and differences between individual plans for these structures (B).

**Figure 4:**
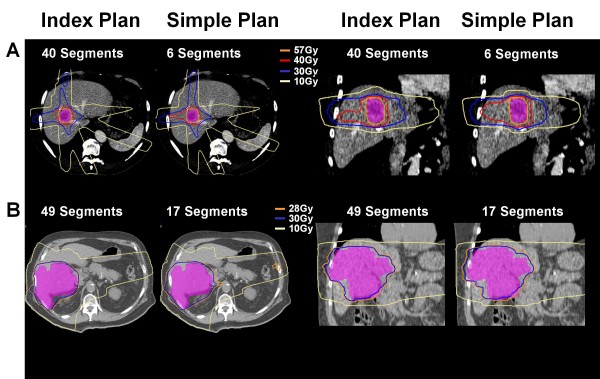
**Axial (left panels) and Coronal (right panels) slices of acceptable index and simple IMRT, showing the six fraction, lowest isodose covering 0.5 cc PTV (orange), the 30 Gy isodose (dark blue) and 10 Gy isodose (beige) surrounding the PTV (pink colorwash)**. Examples are shown of a small lesion typical of liver SBRT (A), where loss of dose conformation of higher isodoses may have larger effects on normal tissue function, as compared to a larger lesion near bowel treated to lower doses (B).

Maximum dose delivered to the heart (mean 25.3 Gy vs. 24.5 Gy, p = 0.048) and ribs (mean 38.7 Gy vs. 37.7 Gy, p = 0.044) was significantly higher for simple IMRT (figure 3) but no other statistically significant differences were seen in other OARs, liver Veff, biological liver NTCP or mean liver dose. Examples of simple IMRT and index IMRT plans are shown in figure 4.

**Figure 5:**
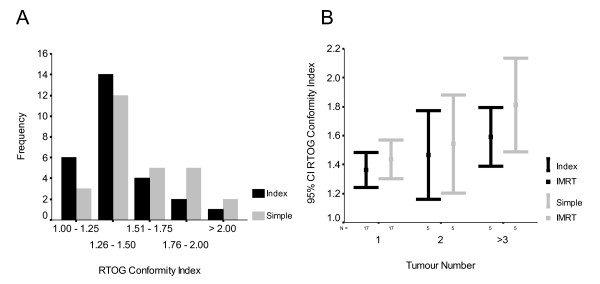
Distribution of RTOG Conformity Index depending on IMRT plan (A) and the 95% confidence interval and mean values shown for different number of tumours and IMRT plan (B).

RTOG CI was significantly higher (poorer dose conformity) for simple IMRT than index IMRT (mean 1.52 vs. 1.42, p = 0.001, figure 5a) with similar differences for plans with prescription dose ≥ 42 Gy (mean 1.33 vs. 1.45, p = 0.026) or < 42 Gy (mean 1.52 vs. 1.60, p = 0.007), demonstrating reduced conformality in the simple IMRT plans. This was also reflected in larger isodose volumes outside PTV for 42 Gy (mean 74 vs. 63 mL, p = 0.025), 30 Gy (mean 364 vs. 323 mL, p = 0.003) but not for 1 Gy (mean 8220 vs. 8271 mL, p = 0.517) or integral dose (69.1 J vs. 68.7 J (p = 0.374)). Using multivariable linear regression, the sole factor that statistically correlated with a higher RTOG CI in simple and index IMRT was number of tumours in the liver (p = 0.004, adjusted R^2 ^= 0.26 and p = 0.047, adjusted R^2 ^= 0.115 respectively) as seen in figure 5b.

### Plan Complexity

Simple IMRT had a significantly reduced complexity compared to the index IMRT using the complexity metric (mean 0.63 vs. 0.51, p < 0.001). Only number of MUs had a consistent correlation with the complexity score using forward linear regression (R^2 ^= 0.52 for whole group, 0.51 for simple plans and 0.56 for index plans, p < 0.001).

## Discussion

This study investigated simplification of IMRT planning for a heterogeneous group of liver cancers (e.g. with high tumour volume and location variability) by reducing the number of IMRT segments and measuring the impact on dose to target and normal tissue volumes. To allow a direct comparison of the effects of reduced segment IMRT plans compared to index, more complex, IMRT plans, similar IMRT planning parameters were used for both situations (i.e. number of beams and angles, minimum segment size and minimum segment monitor units), while only the IMRT segment number was adjusted for plan optimization. Beam angles were chosen based on the angles used clinically for each patient, removing the impact of beam number and beam angle from the comparison. All liver cancers in this study were able to be planned using 30 or fewer segments without exceeding normal tissue constraints while maintaining PTV coverage, showing that it is feasible to treat patients with liver cancer using IMRT with relatively few beam segments. The main compromise seen when using fewer planned segments was a loss in the plan conformity, resulting in higher doses of radiation being delivered to some normal structures. This is likely to be more important as the prescription dose increases (e.g. small typical SBRT tumours treated with doses >48 Gy in 6 fractions); in this setting use of more treatment beams/angles is likely to be more beneficial than only increasing IMRT radiation modulation in an attempt to improve the dose conformity and reduce the potential for undefined late toxicity in high dose regions. These were the minority of cases studied here, as the larger more complex tumours, most likely to benefit from IMRT often have their prescription dose limited by normal tissue limits[4,7]. In these more challenging liver cancer cases, developing a segmented CRT plan manually is not efficient. Use of IMRT with few segments can potentially maximize the benefits of treatment planning efficiency and improved dose conformity without the increased sensitivity of complex IMRT to geometric and dosimetric uncertainties. This may potentially result in the best balance between the benefits of CRT using few numbers of segments and complex multiple segmented IMRT.

The main motivation for studying less complex IMRT in this study was to reduce the negative impact of dosimetric and geometric uncertainties associated with more complex IMRT, in the upper abdomen. Potentially this can also reduce the risk of errors in calculating radiation dose and consequent time taken to perform quality assurance of more complex IMRT plans. Delivered doses are less well correlated with planned doses in the presence of uncertainties, with larger differences in delivered doses expected with more complex IMRT and hypofractionated radiotherapy[17,19]. Reducing segment number should reduce some of the negative impact of these uncertainties. More complex IMRT plans are also expected to have less dosimetric accuracy than simple IMRT plans, with uncertainties in delivered doses of up to 13%[16]. We hypothesize that there will be more concordance between delivered and planned doses with simple liver IMRT. This would also be broadly applicable to other treatments with IMRT where geometric and dosimetric uncertainties are larger (e.g. lung and other upper gastrointestinal tract malignancies). Future work will quantify the changes in delivered doses, accounting for organ motion and residual setup error, in simple versus complex IMRT.

Other benefits of using fewer segments in IMRT include reduced treatment time, associated with improved patient comfort and less potential for intra-fraction error, and reduced MUs, resulting in less leaf leakage, and potentially less risk of second malignancy[13]. In this study, reducing the number of segments reduced the MUs modestly, by 10%, far less than the two-to-three fold increase in MUs with the use of IMRT versus CRT, for other sites[13]. None-the-less, this reduction does improve treatment efficiency[11,27] and may also reduce risk of intra-fraction geometric uncertainty that can be increased by longer treatment time in SBRT treatments[28].

Several groups are exploring other methods to reduce IMRT complexity including use of planning algorithms to control number of segments, size and weighting while optimizing the beam intensity (i.e. direct aperture optimization)[21], allowing similar plan quality while using fewer segment numbers. Other methods used to reduce IMRT complexity include IMRT plan optimization using hybrid CRT/IMRT treatments[29], algorithms to smooth intended radiation fluence[30], and use of modulation penalty cost functions[31]. However these methods may be more difficult to implement in clinical practise. Determining and accounting for geometric uncertainty (i.e. multiple instance geometric approximation)[32] within IMRT planning may eventually allow for plans that are more robust to geometric uncertainties. Finally, minimization of residual uncertainties, with IGRT and breathing motion management, is a recommended strategy to reduce the potential discrepancy between planned and delivered doses, in simple and complex IMRT. Given the rapid advances in technology in radiotherapy delivery, that facilitate delivery of highly conformal radiation therapy, there is a need for well controlled prospective studies to be performed to evaluate the potential benefits or detriments of new technologies and altered fractionations, particularly with respects to the accuracy of the dose delivered, requirements for plan quality assurance and potential toxicities. Given the uncertainties of complex IMRT dose delivery in the liver, the current standard practice at our centre for clinical liver IMRT plans is to aim to use less than 20 beam segments per plan.

## Conclusions

Reducing the number of beam segments is a simple strategy widely available to reduce cancer IMRT plan complexity. Reducing number of beam segments can be performed without a significant detriment in target coverage or normal tissue sparing for liver IMRT for the majority of patients. Reduction of complexity did lead to a reduction in plan conformity without exceeding normal tissue dose objectives. The impact of using fewer beam segments on IMRT plan robustness to residual geometric uncertainties will be investigated in future studies.

## Competing interests

Mark Lee, Tom Purdie and Cynthia Eccles have no conflicts of interest.

Laura Dawson has research funding from Elekta Oncology Systems (within the past 2 years) and Bayer (active). Michael Sharpe is a research collaborator and consultant to Philips Medical Systems and Elekta Oncology Systems and a research collaborator to Raysearch Laboratories AB.

## Authors' contributions

MTL conceived and drafted the manuscript, LAD drafted and revised the manuscript, all authors read and approved the final manuscript.
